# Biochemical responses of juvenile European sturgeon, (*Huso huso*) to a sub-lethal level of copper and cadmium in freshwater and brackish water environments

**DOI:** 10.1186/2052-336X-11-26

**Published:** 2013-08-05

**Authors:** Saeed Zahedi, Arash Akbarzadeh, Maryam Rafati, Mahdi Banaee, Heshmat Sepehri moghadam, Hadi Raeici

**Affiliations:** 1 Department of Fisheries, Faculty of Marine and Atmospheric Sciences, University of Hormozgan, Bandar Abbas, Iran; 2 Department of Natural Resources, Savadkooh Branch, Islamic Azad University, Savadkooh, Iran; 3 Department of Aquaculture, Natural Resource and Environmental Faculty, Behbahan Khatam Alanbia University of Technology, Behbahan, Iran; 4 Department of Agriculture, Payam Noor University, Mashhad, Iran

**Keywords:** Copper, Cadmium, Freshwater, Brackish water, Biochemical responses, *Huso huso*

## Abstract

In Caspian Sea basin, sturgeons spend the larval and juvenile stages in freshwaters of rivers and then, they migrate to brackish waters of the sea where they grow and mature. With regard to the elevation of the metal concentrations in coastal waters and sediments of the Caspian Sea and its adjacent rivers, it is likely that juvenile sturgeon are exposed to sub-lethal levels of metals during seawater entry process. We compared the biochemical responses of juvenile European sturgeon, (Beluga, *Huso huso*) exposed to a sub-lethal level of copper (Cu, 20 μg/L) and cadmium (Cd, 300 μg/L) in freshwater (FW, 0 ppt) and brackish water (BW, 11 ppt) for seven days. The results showed that the levels of plasma glucose increased significantly in BW and in all metal exposed groups. Also, plasma cortisol concentrations showed significant increases when juveniles were exposed to BW, Cu(FW/BW) and Cd(BW). The activity of liver superoxide dismutase (SOD) decreased significantly in BW compared with FW. Moreover, Cu and Cd exposure enhanced the activity of SOD in BW, while SOD did not show any changes in FW. The levels of tissue and plasma proteins as well as plasma triiodothyronine (T_3_), thyroxine (T_4_) and liver Catalase (CAT) activity remained constant when animals were exposed to Cu/Cd in both FW and BW environments. Our data indicate that exposure of juvenile beluga to BW stimulated the general biochemical responses of stress such as cortisol and glucose, while sub-lethal exposure to Cu and Cd caused oxidative stress in BW environment but not in FW.

## Background

Metals are important groups of non-degradable pollutants of environment and different anthropogenic activities as well as natural processes can lead to accumulation of these cumulative pollutants in the aquatic bodies [1,2]. Chronic contamination of freshwater and marine environments by metals, like copper (Cu) and cadmium (Cd) is frequently reported and it is considered as a severe and pervasive concern [3-5].

Exposure of aquatic organisms even with sub-lethal concentrations of metals may cause biochemical and ionic disturbances or adaptive responses in blood and tissues [6,7]. In fact, various genetic, physiological and biochemical factors and behavior of fish could change as sensitive biomarkers when they are exposed to sub-lethal concentrations of metals, like Cu and Cd [8-11]. Exposure of aquatic organisms to metals may result in production of reactive oxygen species (ROS) such as hydrogen peroxide, superoxide radicals and hydroxyl radicals leading to impairment of normal oxidative metabolism and oxidative stress [12,13]. In response to oxidative stress, the antioxidant defense system of aquatic organisms is activated [14-17]. The antioxidant system include various enzymes such as superoxide dismutases (SOD) which catalyze the dismutation of superoxide radical to oxygen and hydrogen peroxide as well as catalase (CAT) and glutathione peroxidase (GPx) which act to degrade hydrogen peroxide [13].

Available data show that the toxic effects of metals depend on a range of biotic and abiotic factors [18]. Among the abiotic factors, salinity has a negative effect on metal toxicity and accumulation, so that its increase reduces the metal toxicity [13,18,19]. Salinity affects the metal bioavailability and uptake and its subsequent toxicity through competing with metal ions for binding to biological molecules [1,20]. Moreover, water salinity increase is associated with the increased ROS generation in organism’s body [21]. Thus, the antioxidant enzyme activity alterations have been reported during water salinity changes [22,23]. This is in particular important for juveniles of anadromous fish when migrating from freshwater to seawater. Anadromous fish must develop complex osmoregulatory mechanisms to survive successfully in both the estuaries and the sea during their seawater entry process [24,25]. In this regard, it is important to examine more realistically the toxic effects of metals in different environments in order to estimate the consequences that fish face during downstream migration.

In Caspian Sea basin, the juveniles of sturgeons migrate from freshwaters of rivers to brackish waters of the sea where they spend most of their life cycle there. European sturgeon, (Beluga, *Huso huso*), is one of the most important sturgeon species in Caspian Sea that its generation is critically endangered [26]. Increasing pollution of the Caspian Sea is one of the major threats to the survival of fish. Since, elevation of the metal concentrations in coastal waters and sediments of the Caspian Sea and its adjacent rivers forming a significant part of the Caspian Sea pollution [4,27-31], so heavy metals can be a potential threat to health of the fish in both freshwater and seawater. In such environments, beluga juveniles may experience transient fluctuations in metal concentrations during downstream migration and seawater entry process. Therefore, the aim of this study is to compare the biochemical responses of juvenile beluga exposed to sub-lethal concentrations of Cu and Cd in both freshwater (FW) and the brackish water (BW).

## Methods

### Fish

The juveniles of beluga used in the present study were obtained from Shahid Marjani Sturgeon Center (Golestan province, Iran), and transferred to the laboratory of Shahid Rajaee Sturgeon Hatchery Center (Mazandaran province, Iran) in May, 2008. Fish were stocked in 2000 L freshwater tanks before start of the experiment. 108 fish (55.4 ± 6.8 g in weight, +4 months in age) were randomly selected and transferred from the stock tanks to experimental ones in June. The weights of the fish used in the experiments were not significantly different. The fish were fed 3% of body weight once a day in the morning (at 9:00–9:30 AM).

### Laboratory exposure

Stock solutions of Cd (2000 mg/L) and Cu (1000 mg/L) were prepared using CdCl_2_.2.5H_2_O (China) and CuSO_4_ · 5H_2_O (Merck, Darmstadt, Germany) in 1 liter of double-deionized water. All stock solutions were stored at 4°C. Before commencing the experiments, the stock solutions were diluted to the desired concentrations with FW (0 ppt) and BW (11 ppt). 18 fish for each treatment (3 replicates) were directly introduced to new tanks containing 300 L of FW and BW. In metal exposure treatments, fish were exposed to nominal Cu and Cd concentrations of 20 and 300 μg/L, respectively in FW and BW for 7 days. During the experiments, the physicochemical characteristics of water were measured daily: temperature, 21.2 ± 0.3°C; pH, 7.9 ± 0.2; hardness, 295 ± 15.8 mg CaCO_3_/L; salinity: 11 ± 0.2 ppt. Aeration of tanks was done by means of air stones attached to an air compressor. Every two days, 90% of water was replaced with fresh medium which stored in supplementary stock tanks to minimize metal loss [15]. During exposure, fish were fed daily at 3% of body weight and they were starved for 24 h prior to sampling.

### Sampling and analysis

Salinity levels of the experimental solutions were measured daily by salinimeter (Tanaka, Japan). Cu/Cd was monitored by inductively coupled plasma optical emission spectro metry (ICP-OES) on daily basis. At the sampling time, fish were removed from each treatment and quickly anaesthetized in clove-essence solution (at 9:00–9:30 AM). After anesthesia, their weight was measured. Blood was drawn from the caudal vein, just behind the anal fin and collected into heparinized syringes and transferred to heparinized tubes held on ice until centrifugation. Immediately after blood collection, liver tissue was taken using clean equipment, rinsed by physiological serum, weighed, frozen in liquid nitrogen and stored at −80°C until further analysis. To obtain plasma, blood samples were centrifuged at 10000 rpm for 3 min (+4°C), aliquoted and were stored in −20°C. The liver was homogenized by homogenizer (TRI-I instrument, England) in 100 mM phosphate buffer (pH 7.4, 1:10, w/v) containing 2 mM EDTA and 150 KIU/mL aprotinin as a protease inhibitor. Homogenates were centrifuged at 10,000 rpm (Beckman, Avanti™ 30, USA) for 45 min (+4°C) and supernatant was used as enzyme source. The glucose and total protein levels were measured using enzymatic colorimetric assay and chemical colorimetric assay kits, respectively (Pars Azmoon, Tehran, Iran). Plasma cortisol, triiodothyronine (T_3_) and thyroxine (T_4_) were assayed with commercial ELISA kits (Diagnostics Biochem Canada Inc, Ontario, Canada). CAT (EC.1.11.1.6) and SOD (EC.1.15.1.1) activities were measured using colorimetric assay kits (Nanjing Jiancheng Bioengineering Institute, Nanjing City, P.R China) in microtiter plate format and using ELISA Reader (Sunrise, Tecan, Austria) for optical density recording. All the assays were performed according to manufacturer guidelines. One unit of enzyme activity is the amount of enzyme that catalyzes the oxidation of 1 μmole substrate per minute. The results are accordingly given as U/mg protein.

### Statistical analysis

Data were analyzed by a one-way analysis of variance (ANOVA), followed by a Duncan’s *post hoc* analysis for multiple comparisons. Differences were considered statistically significant at P < 0.05. SPSS (version 17.0) software was used for the statistical analysis.

## Results

Metal contamination caused no changes in water quality parameters in both FW and BW. The concentrations of Cu^2+^ and Cd^2+^ in water of the metal treatments were 17.6 ± 1.1 μg/L and 281.6 ± 9.4 μg/L, respectively. Exposure of *H. Huso* to metals did not cause any fish mortality within 7 days in both FW and BW.

**Figure 1:**
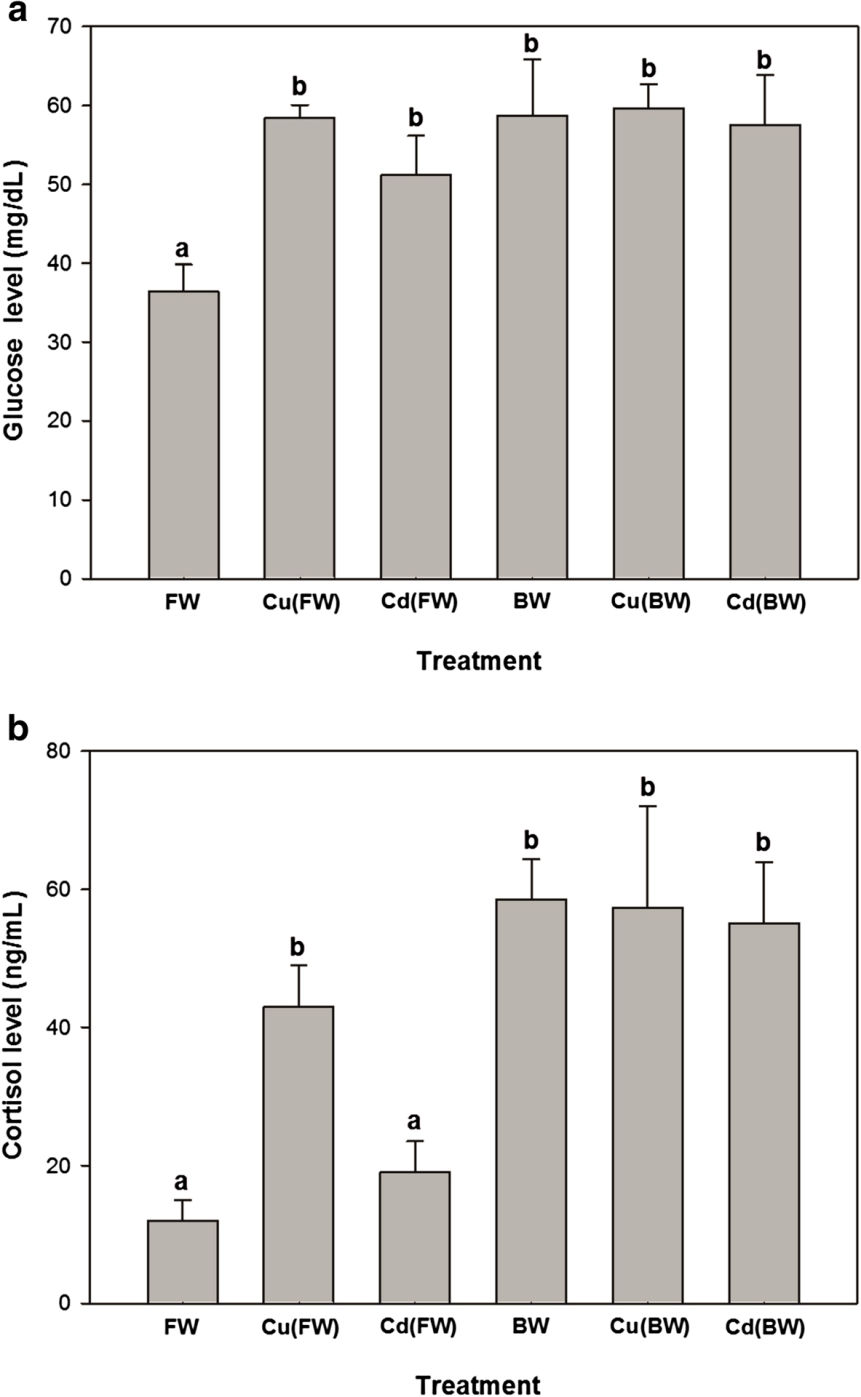
**Glucose (a) and cortisol (b) changes of juvenile European sturgeon, *H. huso *exposed to brackish water (BW, 11 ppt), 20 μg/L of Cu and 300 μg/L of Cd in FW/BW for 7 days (mean ± SE, n = 4–6).** Different letters indicate statistically significant difference among treatments (p < 0.05).

**Figure 2:**
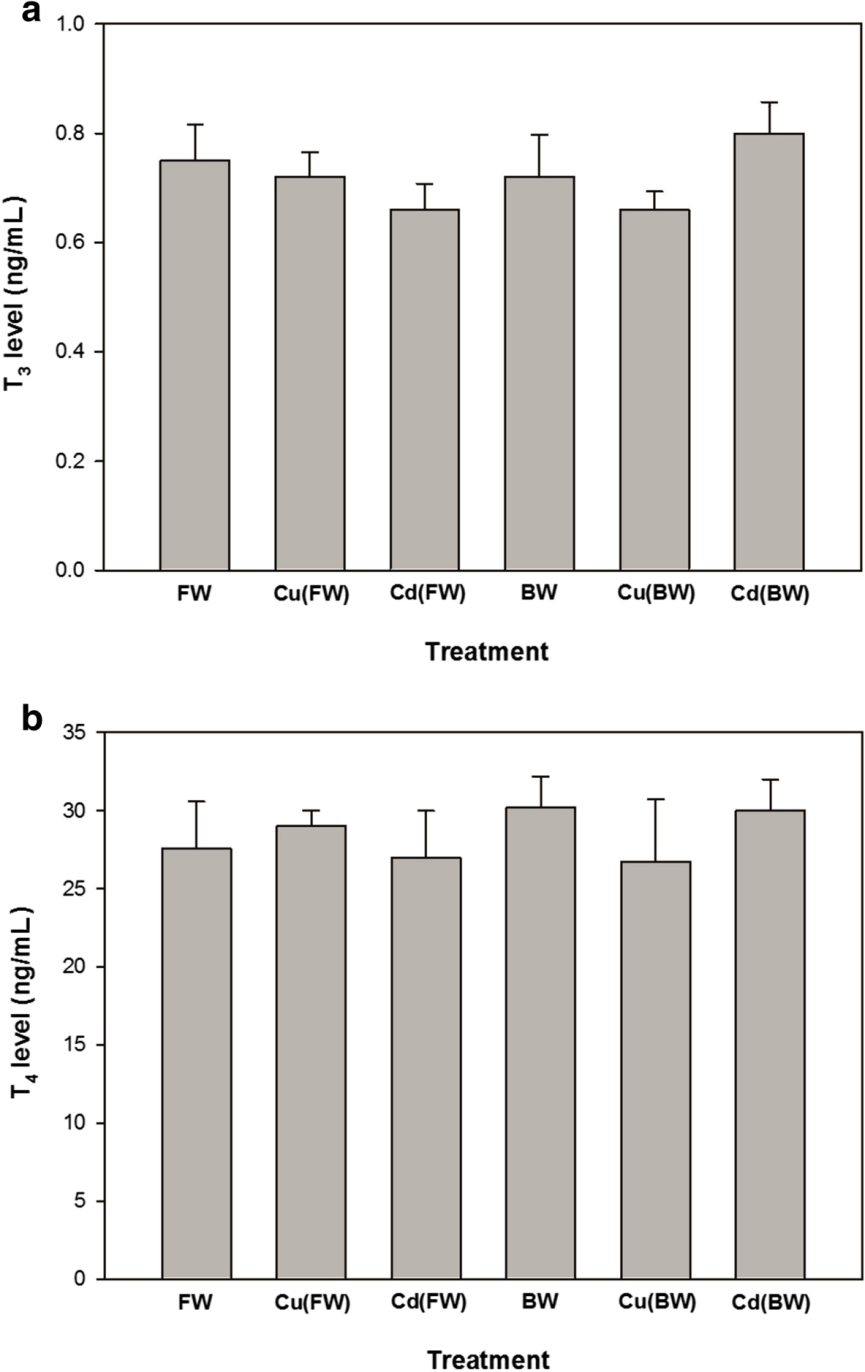
**Concentrations of the plasma thyroid hormones, T_3_ (a) and T_4_ (b) in juvenile European sturgeon,** ***H. huso *** **exposed to brackish water (BW, 11 ppt), 20 μg/L of Cu and 300 μg/L of Cd in FW/BW for 7 days (mean ± SE, n = 4–6).**

Plasma glucose levels increased significantly in the all experimental treatments compared with FW (p < 0.05, Figure 1a). Plasma cortisol levels showed significant increases only when animals exposed to BW, Cu (FW/BW) and Cd (BW) treatments. Also, Cu (BW) and Cd(BW) caused no significant changes in plasma glucose and cortisol compared to BW (Figure 1a, b). The levels of tissue and plasma proteins showed no significant changes when animals exposed to BW or Cu/Cd in both FW and BW environments (Table 1). Also, the levels of plasma T_3_ and T_4_ remained constant in metal exposed and control groups. Moreover, the levels of T_3_ and T_4_ did not differ significantly in FW and BW (Figure 2a, b).

**Table 1:** **Changes in plasma and liver protein in juvenile *H. huso *** **exposed to brackish water (BW, 11 ppt), 20 μg/L of Cu or 300 μg/L of Cd in FW/BW for 7 days**

**Parameters**	**FW**	**Cu(FW)**	**Cd(FW)**	**BW**	**Cu(BW)**	**Cd(BW)**
Plasma protein (g/dL)	1.8 ± 0.2	2.1 ± 0.2	1.8 ± 0.1	2.1 ± 0.1	2 ± 0.2	1.3 ± 0.1
Liver protein (mg/g)	154.2 ± 15.3	167 ± 15.1	167.2 ± 14.1	172.4 ± 27.9	172.8 ± 20.8	137 ± 11.2

Data are presented as mean ± SEM, n = 4–6. Data was analyzed through one-way ANOVA besides Duncan comparisons.

**Figure 3:**
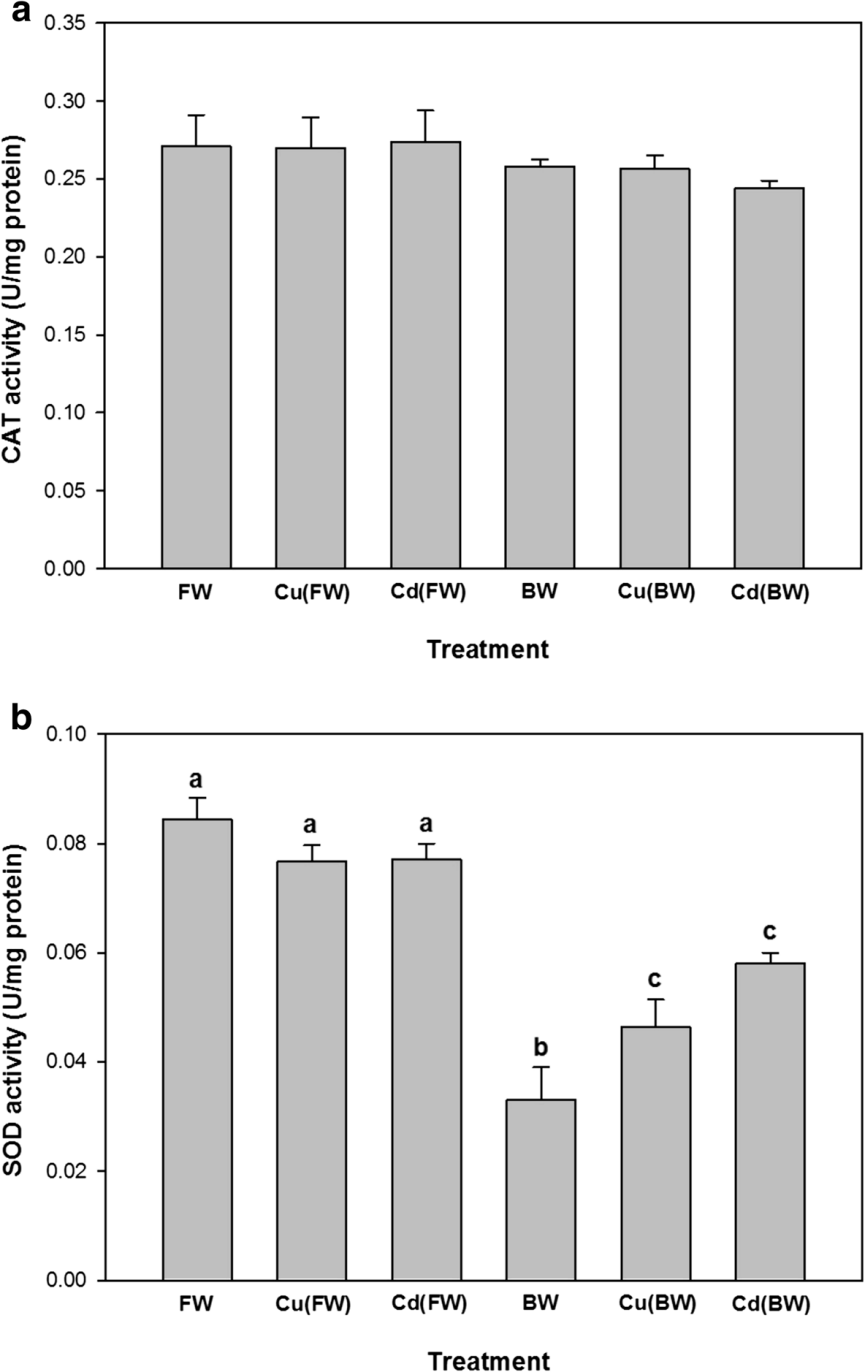
**Liver CAT (a) and SOD (b) activities (U/mg protein) of juvenile European sturgeon, *H. huso *exposed to brackish water (BW, 11 ppt), 20 μg/L of Cu and 300 μg/L of Cd in FW/BW for 7 days (mean ± SE, n = 4–6).** Different letters indicate statistically significant difference among treatments (p < 0.05).

The CAT activity did not differ significantly following BW and metal exposures (p > 0.05, Figure 3a). In contrast, the activity of SOD decreased significantly in BW compared to FW (Figure 3b). Moreover, Cu and Cd exposure enhanced the activity of SOD in BW, while SOD did not show any significant changes in FW.

## Discussion

Exposure of juvenile European sturgeon to BW and a sub-lethal level of Cu(20 μg/L in FW/BW) and Cd(300 μg/L in FW/BW) for 7 days significantly enhanced the levels of non-specific stress response like plasma glucose and cortisol. On the contrary, salinity and metal exposure appeared to have no effect on the other biochemical parameters like plasma/tissue proteins and plasma T_3_ and T_4_. Although, hepatic activity of SOD was clearly lower in BW compared with FW, a significant elevation in SOD activity was observed during Cu(BW) and Cd(BW) exposure. Unlike SOD, the levels of CAT remained unchanged during BW and Cu(FW/BW) and Cd(FW/BW) exposures.

As an ion-regulatory hormone, cortisol is considered a primary indicator of stress response [32]. It is well known that salinity and metal exposures enhance the cortisol levels in fish [8,33-35]. The effects of salinity on fish ion regulation may be the reason for high synthesis and plasmatic levels of cortisol observed in BW and Cu(BW)/Cd(BW) treatments. Generally, osmo-ionic disturbance activates the hypothalamo-pituitary-interrenal axis and subsequent cortisol secretion which stimulate Na^+^, K^+^-ATPase activity [36,37]. In addition, cortisol induces the plasma glucose levels by induction of gluconeogenesis and glycogenolysis for supplying the new energy demand [38,39]. It has been known that the levels of plasma cortisol and glucose usually correlate to each other [40,41]. During metal exposure, the glucose level usually increases but it starts to decline to its initial level on other days of the exposure [33,42]. In contrast, plasma glucose concentrations of the present study remained high even after 7 days of exposure. Similarly, some investigations have shown such trend in plasma glucose levels after aqueous metal exposure [8,43]. It means that juvenile *H. huso* could not adapt themselves to new environments after 7 days of exposure.

Findings of the present study also showed that Cu/Cd exposure in both FW and BW had no significant effect on plasma/liver protein contents. It should be emphasized that tissue protein contents are suitable biomarkers for metal-induced stress, but a consistent trend has not been observed among different studies, and the literature contains several points of conflict [44-46]. It has been stressed that carbohydrates represent the immediate energy precursors for fishes exposed to stress condition, while proteins are spared during chronic period of the pollutant stress [47]. Exposure duration or sampling time (7 days) might affect the obtained results. Juvenile carbohydrates/lipids were probably sufficient for supplying of extra energetic demands during metal exposures, so fish had no need to mobilize proteins for energetic purposes.

Similar to protein contents, the levels of plasma T_3_ and T_4_ did not change in both salinity and metal exposures. It is implicated that thyroid hormones play important roles in fish development, downstream migration and seawater tolerance [48-51]. Our data do not coincide with the results reported by other researchers who have issued plasma T_3_ and T_4_ alterations related to salinity and metal exposures [8,52]. However, results of the present study may suggest that the employed concentrations of salinity (11 ppt) and Cu/Cd could not affect thyroid function and thyroid hormone signaling in beluga juveniles. A number of studies have noted that plasma thyroid hormone levels may be a poor predictive indicator of disruption of the thyroid axis [53-55].

Exposure of *H. huso* juveniles to BW for 7 days resulted in a significant reduction in liver SOD activity compared with FW, but not CAT activity levels. Consistent with the obtained results, a study on *Acipenser naccarii*[23] showed that hepatic CAT and SOD decreased significantly, as environmental salinity increased from FW to 35 ppt. They related decreased SOD and CAT activities to the elevated amount of hepatic protein. On the other hand, increased salinity from freshwater to seawater did not lead to any significant changes in the antioxidant activity of SOD and CAT in a euryhaline teleost *Fundulus heteroclitus*[13]. We assume that different antioxidant activity responses during salinity exposure are related to different osmo-regulation physiology among fishes.

An increase in the liver SOD activity was detected in juveniles exposed to Cu and Cd in BW. However, Cu(FW)/Cd(FW)-exposed fish showed no significant differences in SOD activity. These results indicate that oxidative stress is probably increased during metal exposure in BW and only manifested in SOD activity. To cope with oxidative stress caused by metal exposure, the antioxidant defense system of aquatic organisms is activated [14-17]. Changes in antioxidant enzyme activity especially for those of CAT and SOD have been reported during metal exposure in fish [14,16]. It has been suggested that SOD is more involved in protection against destruction caused by ROS compared with CAT [56]. SODs are a group of metalloenzymes that plays a crucial antioxidant role and constitutes a defense system against the natural or chemically induced production of ROS [21,22]. Accordingly, the SOD activity increased in three-spined stickleback *Gasterosteus aculeatus*, during the first week of Cu exposure [14]. The stimulation of antioxidant parameters has been reported in the liver of *Oreochromis niloticus* exposed to chromium (Cr) and lead (Pb) when salinity increased [57].

## Conclusions

The results of the present study showed that exposure of juvenile European sturgeon to BW and a sub-lethal level of Cu(FW/BW) and Cd(FW/BW) enhanced the plasma levels of non-specific stress response like plasma glucose and cortisol. Moreover, the obtained data showed that hepatic activity of SOD increased clearly in fish exposed to Cu and Cd in BW, probably due to the increased oxidative stress. These results indicate that even a small sub-lethal level of the tested metals can be stressful for juvenile European sturgeon.

## Competing interests

All authors declare that they have no competing interest.

## Authors’ contributions

SZ was the main investigator, designed and performed the study and drafted the manuscript. AA supervised the study. MR, MB and HSM were advisors of the study. HR helped in the statistical analysis. All authors read and approved the final manuscript.
